# Evaluation and Utilization of Aged Bacteria in MICP Technology

**DOI:** 10.3390/ma19061122

**Published:** 2026-03-13

**Authors:** Masaharu Fukue, Zbigniew Lechowicz, Catherine N. Mulligan, Seiichi Takeuchi, Hidekatsu Takeuchi

**Affiliations:** 1 Japanese Geotechnical Association for Housing Disaster Prevention, 1622, Oshikiri, Shimizu-ku, Shizuoka 424-0008, Japan; fukue.masaharu.h@tokai.ac.jp; 2 Department of Geotechnical Engineering, Institute of Civil Engineering, Warsaw University of Life Sciences, Nowoursynowska 159, 02-776 Warsaw, Poland; zbigniew_lechowicz@sggw.edu.pl; 3 Department of Building, Civil and Environmental Engineering, Concordia University, 1455 de Maisonneuve Blvd. W., Montreal, QC H3G 1M8, Canada; 4 Fudo Tetra Co., 7-2, Koami-Cho, Nihonbashi, Chuo-ku, Tokyo 103-0016, Japan; seiichi.takeuchi@fudotetra.co.jp (S.T.); hidekatsu.takeuchi@fudotetra.co.jp (H.T.)

**Keywords:** MICP process, optical density (OD), cell viability, carbonate precipitation rate (CPR), soil–biocement solution (BCS) system, apparent viability rate (Rcv), aged microorganisms, diffuse double layer

## Abstract

As a response to aging of cultured urease-producing microorganisms, the blending method was examined to obtain the required carbonate production amount using the apparent viability (Rcv) based on previous research. As a result, a significantly higher carbonate content than the amount of CaCl_2_ 2H_2_O used was produced. Since this trend has been obtained in previous studies, it was judged that carbonate hydrate was formed. As a next step, a penetration test of soil–biocement–liquid (BCS) was conducted to investigate the properties and behavior of the BCS system, taking into account the microscopic properties of the BCS response. The depth distribution of carbonate content (C) was measured by the acid dissolution method of soil sampled from the specimen. It was assumed that the C-profile was formed by adsorption based on the diffuse double layer of microorganisms. It was shown that the amount of precursor-carbonate (precursor CPR), the optical density (OD) of viable bacteria, and the physical amount of soil adsorbed at that position can be estimated from C obtained at the various depths. In addition, the previously obtained formulas among CPR, viable OD, and Rcv shown are briefly explained in this paper.

## 1. Introduction

### 1.1. MICP

Microbial induced carbonate precipitation (MICP) technology uses induced carbonate precipitation, which is a biological carbonate production function, rather than controlled carbonate precipitation. The meaning of “induced” is not intentional, but “by chance”, and does not include what can be done by chance [1]. Therefore, MICP happens to be the name given to the formation of carbonates in secondary reactions.

The latter “controlled” term refers to biologically controlled carbonate precipitation (BCCP), in which organisms control the production of carbonates for their own protection or function. Typical examples of the latter are carbonate minerals such as coral, shell, foraminifer, coccolith, etc. The formation of limestones is due to diagenesis of sedimented carbonate biominerals, but it is not clear whether diagenesis is due to biologically (microbially) induced carbonate precipitation (BICP or MICP). It is interesting that the solidification characteristics of Ryukyu limestone showed a similar trend to the artificial MICP treatment of fine sand [2].

In nature, there are six types of metabolism, i.e., different MICP processes [3]. They are (a) photosynthesis by *Cyanobacteria*, (b) ureolysis by urease-producing bacteria, (c) denitrification by nitrate-reducing bacteria, (d) ammonification by *Myxobacteria*, (e) sulfate reduction by sulfate-reducing bacteria, and (f) methane oxidation by methanogens. These metabolisms have been studied in diverse fields like soil improvement, and soil and water remediation [4,5,6,7,8,9,10,11,12,13,14].

However, many technologies use urease-producing bacteria because it is easier to prepare urea and calcium chloride as materials [15,16,17,18,19,20,21,22]. In addition, there are two typical MICP processes, the Ca^2+^-controlled and bacteria mass (OD)-controlled processes [23]. The former is described as MICP under a relatively low Ca^2+^/biomass (OD) ratio, while the latter is the case when the biomass (OD) is limited. This means that there is an optimum Ca^2+^/biomass ratio, as described later. The previous study revealed that too low OD values cause inhibition and retardation of MICP and that Ca^2+^/OD ratios can control the properties and behavior of CaCO_3_ precipitation [2,21,22]. The problem before this was how OD was defined. In reality, it is often according to a researcher’s own definition or measurements. It is often the basis for non-consensus in research results because the OD does not guarantee any engineering property, but the optical density varies with many factors.

### 1.2. Present Problems

Unfortunately, most experimental data in MICP studies cannot be used to compare research data because it is not known whether the data taken is Ca^2+^-controlled or OD-controlled, and the data of OD are incompatible, as mentioned above.

An essential problem with MICP technology is that it is not easy to assess the ability of the bacteria to be used. In other words, unless the scientific relationship between carbonate production and bacterial content is understood, it is impossible to predict the amount of carbonate production and solidification strength [21,22].

### 1.3. Human-Made Method from Induced Precipitation

In nature, most of the biominerals produced are created in the form of calcite and aragonite mainly in sea areas. On the other hand, carbonates due to artificial MICP treatment are usually inferior to natural biominerals, such as shells, snails, coccoliths, and foraminifers. This difference can be described in terms of control, as opposed to induction. In other words, it means that in nature, the precipitated carbonate biominerals are designed and produced under the control of the organism themselves. The carbonate products are organs necessary for themselves or to protect themselves from external enemies.

Since carbonation by hydrolysis due to urease is a typical induced reaction, it is necessary to raise the reliability of carbonate formation as much as possible. To that end, a new approach has to be examined [2,21,22]. The use of biologically controlled carbonate methods is ideal for this, but currently, there are knowledge gaps. Therefore, in order to enable innovative solutions, the relationships among urea concentration, Ca^2+^, and microbial mass in the hydrolysis of urea, (2) the optimal viable OD and carbonate precipitation rate (CPR) relationships which can be a standard calibration with the conditions of the Ca^2+^ concentration equivalent to the urea molar concentration in BCS, (3) the possibility of CPR as a microbial capacity instead of urease activity, (4) viable OD as a means of uniform evaluation of microbes, and (5) evaluation of aged microbes were examined and reported in previous publications [2,21,22].

### 1.4. Bacterial Controlled MICP (CPR)

The CPR in MICP varies with many factors, which include Ca^2+^ concentration, urea concentration, bacteria mass (optical density: OD), urease activity, temperature, pH, elapsed time, etc. [2,20,21,22,24,25]. The MICP process consists of two chemical reactions, which are hydrolysis due to catalysis with urease and carbonation due to its product, CO_2_. These two reactions do not occur necessarily and depend on the surrounding environment. Therefore, in terms of MICP technology, it is important to design a reaction environment that is optimal without waste.

The problem is the quality and quantity of bacteria and how to evaluate them. In fact, this problem is likely to continue as long as the quantity and quality of microorganisms are not included in the MICP formula. In other words, it requires a fusion of chemistry and biology.

Optical density (OD), which is assumed to be representative of bacterial concentration in BCS, has been used as a prime factor of CPR. Most studies showed that the higher the OD values, the greater the CPR [13,24,26]. However, satisfactory analysis results were not always obtained because of other effects. Recently, it was found that OD-CPR relationships were more complicated due to the effect of time, i.e., linear or non-linear, and Ca^2+^/OD ratio effects, i.e., inhibition or retardation effects [2,21,22]. If the concentration of Ca^2+^ is relatively low and the OD is sufficient, the CPR (M) is constrained by the Ca^2+^ (M) level. This is called Ca^2+^ controlled CPR, while the other type of CPR in this study is called “bacterial controlled CPR”. The ultimate goal of this research is to establish a method that can use microorganisms as a complete engineering material.

## 2. Materials and Methods

### 2.1. Microorganisms Used

In this study, MICP technology was used via the infiltration method of liquid biocement materials consisting of urease-producing bacteria. Bacteria strain NO-A10 used for the experiment was supplied by Life Engineering, Co., Japan, as described by Fukue et al. [21,23].

#### 2.1.1. Rcv Value of Microorganisms Used

**Figure 1:**
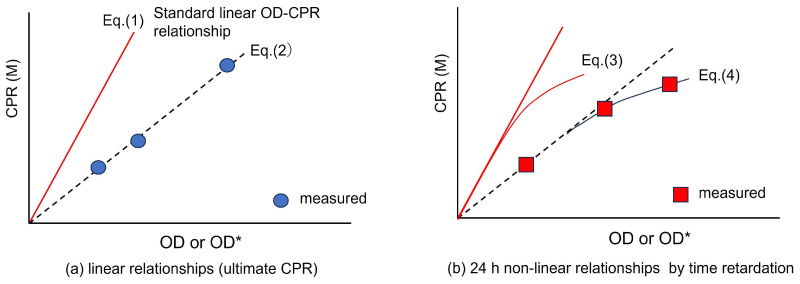
Determination of Rcv using the OD*-CPR fitting line, with different OD* values and constant Rcv, (**a**) without any time limit and (**b**) with 24 h retardation.

First of all, the microorganisms to be used should be evaluated in terms of viability and capacity for carbonate precipitation. In this study, the innovative method of Fukue et al. [21,22] was utilized. The method uses the CPR values determined by using different dilutions of the BCS containing the dummy optical density OD*. The experimentally obtained OD*-CPR measurements can be compared with the corresponding standard OD-CPR relationship, as illustrated in Figure 1. The four equations, linear OD-CPR, linear OD*-CPR, non-linear OD-CPR and non-linear OD*-CPR relationships, were defined in the previous study [21,22], as

CPR = 8.46 OD(1)

CPR = 8.46 Rcv OD*(2)

CPR = 8.46 OD − 17.633 OD^2^ (3)

CPR = 8.46 Rcv OD* − 17.633 (Rcv OD*)^2^ (4)

where Equation (1) indicates the carbonate-producing ultimate ability per viable OD. On the other hand, Equation (2) provides an OD*-CPR relationship due to the low capacity of aged bacteria. In other words, the carbonate purification capacity decreases by the amount of dead bacteria, but the optical density containing the number of dead microorganisms is expressed by OD*. It is noted that Equations (1) and (3) are unique, but Equations (2) and (4) are unlimited with Rcv values [21,22].

Equation (3) shows the OD-CPR relationship when Equation (1) includes a retardation effect 24 h after the start of the carbonation reaction, and Equation (4) shows when both the retardation effect and the effect of microbial degradation are included.

In general, 24 h OD*-CPR relationships are also used in the Rcv test method [21].

The OD*-CPR relationships can be linear or non-linear, depending on the time until measurement of CPR, as shown in Figure 1a,b. If the time is sufficient, the CPR should be the best when the relationship becomes linear, while insufficient time causes retardation.

The determination of Rcv values can be achieved geometrically or by calculation from the ratio of OD/OD* at the same CPR [21]. Thus, in this study, the CPR is the root values for representing the capacity of bacteria. It is apparent that the relationship is given by

Rcv = OD/OD*(5)

This is because the standard OD-CPR relationship was defined as CPR = 8.46 M at OD = 1.0 under normal conditions, such as when the chemical reaction is not inhibited.

Equation (5) shows the conversion of OD and OD*. This allows all data retrieved in OD* to be converted into unified data in OD. As a result, a unified view of MICP has been obtained, and many things have been understood [2,21,22].

#### 2.1.2. Determination of OD* for Blending of BCS

**Figure 2:**
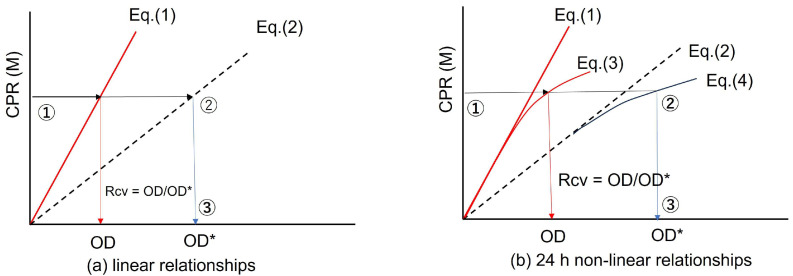
Prediction of target OD* value for the optimal blending of BCS from the target CPR value. ① indicates the target CPR, and ② indicates the position of OD* in the target CPR by OD. ③ indicates the final target OD*.

Though the Rcv can be determined using a dummy OD*, as described in the last section, a target OD* cannot be predicted without the Rcv value of the bacteria. This is because to prepare BCS in a MICP project, a target OD* is needed. The target OD* can be one corresponding to the maximum CPR to be produced. Therefore, the value can be determined easily, as seen in Figure 2a,b. In terms of actual performance, OD* and bacterial mass must be linked in advance. For that, the Rcv of the bacteria must be determined.

Figure 2 is basically similar to Figure 1. However, the objective for use is not the same. Figure 2 illustrates how to find the target OD* for the optimal blending of BCS. In Figure 2, Equation (1) is the unique and linear standard OD-CPR for both Figure 2a,b. The curves in Figure 2b are of Rcv = 1.0 and the Rcv of bacteria to be used. If the OD*-CPR curve (Equation (4) with the Rcv value is obtained, the target OD* can be estimated from the CPR value.

When frozen concentrated bacteria are used for biocement solution, the frozen bacteria are thawed and used to prepare blending of BCS. Thus, in this study, in accordance with the law of conservation of mass, the following equation was formulated using the OD* of BCS and the optical density OD_i_ at the end of microbial culture. In other words, the amount of biomass to be prepared is equal to the biomass to be used.

V_BAC_ = V_BCS_ OD*/(OD_i_ ∙ C_f_) (6)

where V_BCS_ is the volume of BCS and C_f_ is the concentration factor due to centrifuging. In Equation (6), V_BCS_ ∙ OD* is the volume of BCS times the optical density, and (V_BAC_ C_f_) ∙ OD_i_ is the volume of culture times the optical density of the culture. The amount of bacteria usually used is very small, about 1/1000 compared to BCS. In this study, OD_i_ and C_f_ are always assumed to be constants. There are various reasons for this. For example, even if microorganisms are cultured, even in batch, there are often differences in the quality of bacteria. Even after that, it is difficult to take into account the effects of temperature, aging, pH, centrifugation work, environmental changes during transportation, or the effects of glycerol added to rapid freezing. In this technology, we have developed a method that can change a standard OD-CPR relationship by using a dummy OD*. The most important results can be expressed in terms of OD and CPR. However, it is important to note the results cannot be obtained without the roles of OD* and Rcv.

#### 2.1.3. Rcv Values Used

**Figure 3:**
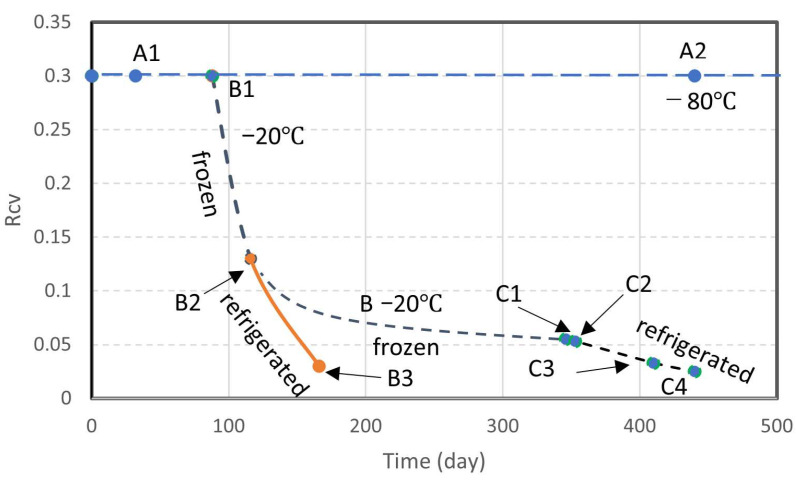
The varied Rcv values of initially rapid frozen bacteria kept at different temperature histories (NO-A10). B1: change from −80 to −20 °C, B2 and C1: change from freezer to refrigerator, i.e., from −20 to 4 °C.

In this study, the bacteria were identified by the Rcv values [21]. Figure 3 shows the Rcv of the NO-A10 strains kept in the freezer at −80 °C and higher temperatures, −20 °C and 4 °C. The results show that at a temperature of −80 °C, the reduction in Rcv, i.e., the quality of bacteria is negligible, but it seems that the greater the temperature increase, the greater the reduction in Rcv. In addition, at a low level of Rcv, its reduction rate was mitigated, as seen in the B line at −20 °C.

In this study, relatively high capability (Rcv = 0.3) and low capability (Rcv = 0.03) were used to examine the present subject. In fact, the concept of Rcv is not widespread and the trial is a new approach. It is noted that the Rcv values were determined according to the designated Rcv test [21].

In Figure 3, bacteria (NO-A10) at the stages of A2 and C4 were respectively used in this study. Note that the capacity of bacteria A1 is about 10 fold higer than that of C4, i.e., 0.3 against approximately 0.03 in Rcv values.

### 2.2. Materials Used

#### 2.2.1. Chemical Agents

The main chemical agents and materials used in this study were urea and calcium chloride monohydrates, which have been common agents used in MICP technology [19,20,27]. The bacteria (NO-A10) are not chemical agents but provide enzymes (urease) as the catalysts. The advantages of using urea are that it is easy to obtain as an ingredient and it can produce a lot of carbonates at once.

#### 2.2.2. Sandy Soils

In order to investigate the characteristics of the soil–BCS system, natural coastal sand was used. The properties of the sand are described in Section 3.3.

### 2.3. Experiments

#### Methods

*Soil system*

*--Hydrolysis in BCS*

The BCS itself is not the binding system but contains mainly suspended materials (bacteria) and solutes (Ca^2+^, Cl^−^ and urea). The bacteria degrade urea by hydrolysis and produce NH_3_ and CO_2_, which are released extracellularly through the cell membranes, Ca^2+^ [28].

The first chemical reaction in MICP is hydrolysis, which has been expressed by

m(NH_2_)_2_ CO + [e] + 2mH_2_O → 2mNH_4_^+^ + mCO_3_^2−^ (7)

where [e] denotes the enzyme, urease, for the hydrolysis of urea. However, there has not been any quantitative expression and definition of [e] that satisfy the necessary conditions for the required CPR in Equation (7), except for the viable OD defined by the previous study [21,22]. Therefore, there have not always been reliable results obtained by using Equation (7) in terms of considering optimization, inhibition, and retardation in MICP. Note that in this study, the quantity of enzyme is expressed by a viable OD.

In the BCS, the CO_3_^2−^, which is produced from CO_2_ released from the inside of cell, reacts with Ca^2+^. In general, in BCS, the cation Ca^2+^ is attracted to the surfaces of bacteria that are generally negatively charged. The theoretical explanation can be made by the diffuse double layer theory. Therefore, the calcite produced by BCS forms crystals with bacteria as its nucleus and the precursors of calcite are ACC and vaterite [29].

The BCS must be blended to ensure that the carbonates produced are under optimal conditions. The optimum condition must be technically and economically optimal or close to it. It must be intended for a standard linear OD-CPR relationship (Equation (1)), not Ca^2+^ control or microbial control.

Equation (1) shows that the amount of carbonate production is determined by the OD, but in terms of the Ca^2+^ concentration required for this, it is the following equation:

Ca^2+^ (M) = 8.46 OD (8)

Equation (8) is the condition to provide optimal CPR in Equation (1). The Ca^2+^ can ideally react with CO_3_^2−^ in BCS. The CO_3_^2−^ is supplied as a result of the hydrolysis of urea in bacterial cells [28], though the first products are NH_3_ and CO_2_. The products are released into the BCS, which are present outside the cell membrane. The cell membrane surface is negatively charged, and during BCS, Ca^2+^ is attracted to form a diffuse double layer and carbonates are produced within this layer. In general, ACC, monohydrated calcite, vaterite, and/or calcite are produced. Aragonite production may require the presence of magnesium ions [23]. Since CO_3_^2−^ originates here, CO_2_ is released from inside cells, and carbonates are easily formed in the diffuse double layer of bacteria. In general, the nuclei of the carbonate are considered to be bacteria.

The formation of the diffuse double layer typically lowers the zeta potential and reduces the repulsion between bacteria, and the aggregation of suspended bacteria begins to occur. In addition, they are adsorbed to the side of the glass test tubes and soil particles in soil pores. Note that the glass surface may have positive charges, while soil particles have negative surface charges. These phenomena are due to aggregates of negatively charged bacteria and Ca^2+^ cations.

Whether or not carbonate precipitation occurs is dependent on the Ca^2+^/OD value. In addition, it is convenient that the ratio Ca^2+^/OD is constant for various OD values. This means that when BCS is diluted or concentrated, its concentration ratio remains unchanged, and optimal conditions are maintained. It is important to note that if the Ca^2+^/OD ratio is greater than 8.46 M, the inhibition in carbonate precipitation occurs. Therefore, it is suggested that the Ca^2+^/OD should be a little less than 8.46 M. It is also important to note that OD is a viable optical density but not OD*.

The optimum OD can be determined from the target CPR, using Equation (1). When the target CPR is 0.5 M, then the OD required is 0.5/8.46 = 0.059. The Ca^2+^ required in BCS is 0.059 M. Thus, the biomass (amount of microorganisms) required in the hydrolysis reaction in MICP is given by the OD value.

The OD* in the preparation of BCS can be easily obtained by Equation (2). Then, for example, OD* = OD/Rcv = 0.059/Rcv. When Rcv = 0.3, OD* = 0.197.

Finally, Equation (6) is needed to determine the biomass required in BCS. For example,

Biomass (L) = V_BAC_ = V_BCS_ OD*/(OD_i_ ∙ C_f_) = [2000 (L) ∙ 0.197]/(4.0 ∙ 250) = 0.394 L (9)

Thus, 0.394 L of bacteria is required to produce 2 m^3^ BCS for 0.5 M CaCO_3_. In this study, to produce 0.25 M CaCO_3_, the optimum blending design is examined with a very low level of Rcv, i.e., values lower than 0.033.

*CPR Predicted*

Since the bacteria required for optimal blending can be predicted in the procedures described earlier, the CPR is theoretically estimated as

mCa^2+^ + mCO_3_ → mCaCO_3_ ↓ (10)

where m is the molarity. The CPR to be predicted by Equation (10) occurs as a continuing reaction of Equation (7).

*Carbonate content*

The carbonate content C was defined as

(11) $${C=(C}_{m}-C_{i})\cdot 100\;(\%)$$

where C is the carbonate content in MICP, C_m_ is the measured carbonate content, and C_i_ is the initial carbonate content. To obtain the mass of carbonate, a calcite-acid reactor was used [23]. The advantage of this method is that in the case of sandy soil, a small amount of soil sample (a few grams) is sufficient. Therefore, many samples can be tested at once. The disadvantage is that it cannot be used on calcareous soils. In this study, Equation (6) is examined experimentally using different Rcv values, i.e., variously aged bacteria. In addition, the morphological feature of CaCO_3_ produced is observed with a digital microscope.

An innovative approach to the BCS-soil system is proposed as a future study in MICP technology, which includes the adsorption and crystallization behavior of physicochemical interactions among solutes, bacteria (suspended solids), and soil particles during infiltration.

## 3. Test Results

### 3.1. Rcv and CPR

The preparation of optimally blended BCS was demonstrated in the Methods section. The formulation of bacterial mass required for BCS was expressed in Equation (6). In this study, optimal-blending CPR values of BCS containing 0.25 M Ca^2+^ are examined using aged, concentrated bacteria with low Rcv values, i.e., lower than 0.03. The results are presented in Table 1a. For comparison, the case where Rcv is 0.3 is presented in Table 1b. The classification by R_CV_ is shown in Figure 3. The sample in Table 1a is shown in C4 in Figure 3, and Table 1b is shown in A2.

Table 1 indicates that the ultimate CPR values of most samples showed higher values than 0.25 M calcium carbonates in the use of 0.25 M CaCl_2_. This cannot be explained by a simple chemical reaction. Our blending of biocement solution was targeted for 0.25 M calcite. Therefore, the CPR was calculated assuming the production of calcite. As a possibility, the phenomenon has been observed experimentally in the MICP process [21] and may be explained only by the formation of hydrated calcite [30,31,32,33]. Regarding the hydration of calcium carbonate, CaCO_3_ ∙ H_2_O and CaCO_3_ ∙ 6H_2_O have been relatively well known, and there may be examples of other hydrated calcites as well. If the carbonates produced using 0.25 M Ca^2+^ are all monohydrated calcite, the molecular weight of the monohydrated calcite should be 295, which is likely some or all of the specimens shown in Table 1. However, at present, no conclusion has been reached.

### 3.2. Morphological Observation by Digital Microscope

**Figure 4:**
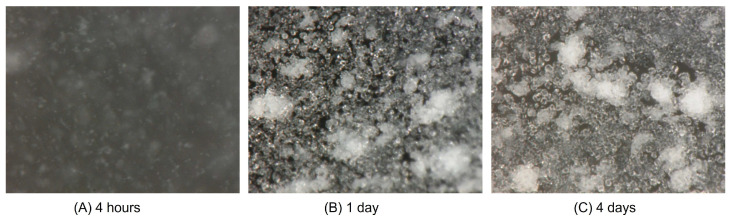
Calcium carbonate precipitated in the glass tube No. 50.

**Figure 5:**
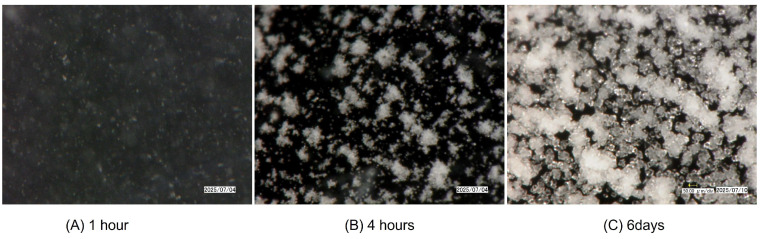
Calcium carbonate precipitated in the glass tube No. 56.

Using a digital microscope, the time-series carbonate formation of all BCS samples was observed. Figure 4 (No. 50) and Figure 5 (No. 56) show faster and larger amounts of carbonate precipitation in relatively high OD* values. The difference between each is, for example, due to the glycerol homogeneity issues during flash freezing or insufficient agitation in BCS preparation that can cause errors which cannot be ignored in small-scale tests and experiments. In any case, the carbonate formation status seen from each CPR and the photo seem to be consistent.

### 3.3. Application to Soil and BCS System

#### 3.3.1. Present Problems in Treatment of Soils and BCS System

In MICP technology, carbonate content is a strong factor for judging soil improvement. However, it is very difficult to predict the profile of carbonate content because of the infiltration properties and behavior of BCS, which can vary in terms of types of soil (dry density, permeability, texture of particles, etc.), interactions and adsorption properties among soil particles, bacteria and solutes (Ca^2+^ and CO_3_^2−^), and crystallization properties of CaCO_3_. In addition, the interactions, adsorption, and crystallization behavior are time-, temperature-, and pH-dependent processes.

Under this situation, it is obviously difficult to infer the osmotic behavior from the prepared BCS. For example, the surface charge of the adsorption material and the associated infiltration behavior may be different depending on the time from preparation to infiltration of BCS. This is because dispersion, aggregation, crystallization, and adsorption are all functions of time, respectively, during infiltration. In addition, the permeability of the soil, the drying density, and the surface texture of the particles also have an impact. It may be better to consider how to predict behavior of the MICP characteristics of microorganisms from a multifaceted experimental plan rather than predicting behavior.

#### 3.3.2. Formulation of Relationship Among Carbonate Contents, CPR, and OD

It is important to realize that when the original injected BCS penetrates into the soil, the substance in the liquid is adsorbed by the soil particles, so the concentration of the substance gradually decreases with infiltration. Therefore, it is extremely difficult to evenly apply the material in the MICP infiltration method. In order to carry out the construction as evenly as possible, it is necessary to use the infiltration characteristics of BCS that change with the soil quality, the estimation of the carbonate formation distribution, and the method of repeated infiltration. On the other hand, the optimal composition of materials in BCS will reduce the concentration with adsorption, but the proportion will not change, so it is thought that it will be possible to handle it theoretically to some extent.

According to the application of adsorption theory, bacterial adsorption seems to be simply expressed by the Langmuir and Freundlich or modified theories. For example, the adsorption of microorganisms on soil particles similar to the partition coefficient is obtained [34]. However, in MICP, the adsorption of bacteria on soil particles occurs as bacteria–aggregates with Ca^2+^. The initial aggregates may be ACC and vaterite, which change to crystalline CaCO_3_ [35]. Under the ideal blending conditions of the BCS, the complex aggregates may depend on at least the OD and initial Ca^2+^, flow velocity of BCS, and soil dry density.

**Figure 6:**
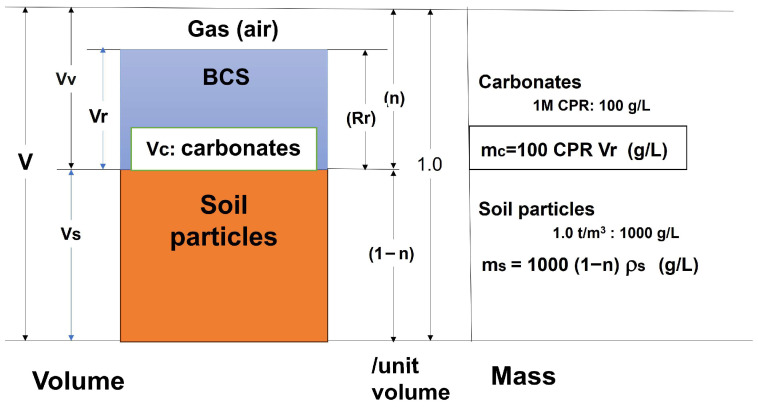
BCS—soil system to illustrate the relationship between carbonate content and CPR.

In this study, a concept based on the CPR depending on OD is used, which is presented by Equation (1), which indicates that CPR is expressed as a function of OD. Therefore, if a CPR value is given, OD is necessarily determined. Note that the opposite is also true. If BCS is infiltrated into soils and saturated with BCS for a certain time, porosity *n* should be used for the volume of BCS. In this case, the relationship between C and CPR is given by

(12) $$C=\frac{m_{c}}{m_{s}}\cdot 100=CPR\;\frac{n}{\left(1-n\right)}\frac{\left(0.1\right)}{\rho_{s}}\cdot 100\left(\%\right)$$

where m_c_ is the mass of carbonates, m_s_ is the mass of soil particles, and ρ_s_ is the density of soil particles (Figure 6). Note that in Equation (12), the infiltration of BCS results in a decrease in the CPR, which will reduce C in Equation (12), because the solute concentration of BCS will decrease due to the adsorption of bacteria and solutes (Ca^2+^ and CO_3_^−^) with the distance of infiltration. In other words, according to adsorption theory, the amount of adsorption decreases as the equilibrium concentration of BCS decreases. Note that the properties and behavior of this process are more complicated in terms of natural solutes such as Mg and other metals in the soil. The aggregation, formation of amorphous materials, and crystallization of calcium carbonates, such as hydrated calcite, vaterite, calcite, and aragonite may occur. However, in this study, these mineralogical properties are not discussed here, but will be treated collectively as calcium carbonate.

In order to consider the unsaturated condition during the curing of crystal growth of calcite, the retention rate (Rr) of the BCS in the soil pores is induced, as

(13) $$C=\frac{m_{c}}{m_{s}}\cdot 100=Rr\cdot CPR\frac{n}{\left(1-n\right)}\frac{\left(0.1\right)}{\rho_{s}}\cdot 100\left(\%\right)$$

##### Example of Application to Fine Sand

Assuming that the retardation effects due to OD^2^ are negligible because of the sufficient passage of time, and using Equations (1) and (13), the relation between C and OD is given by

(14) C=8.46OD·W (%)

where

(15) $$W=Rr\;\frac{n}{\left(1-n\right)}\frac{\left(0.1\right)}{\rho_{s}}\cdot 100\;(\%/M)$$

From Equation (14),

OD = 0.118 C/W(16)

where C is usually measured and W is constant, which can be calculated using constant Rr and soil properties. Thus, OD is proportional to C (z) in the infiltration of BCS, where C (z) means that C is a function of distance (z) from the inlet.

The applicability of Equation (13) was examined by a simple infiltration experiment using a small mold, like a plastic bottle. After 1000 mL of BCS were injected into the sand specimen four times, 1000 mL of tap water were used as a rinse. The conditions of BCS in the infiltration tests are presented in Table 2.

**Figure 7:**
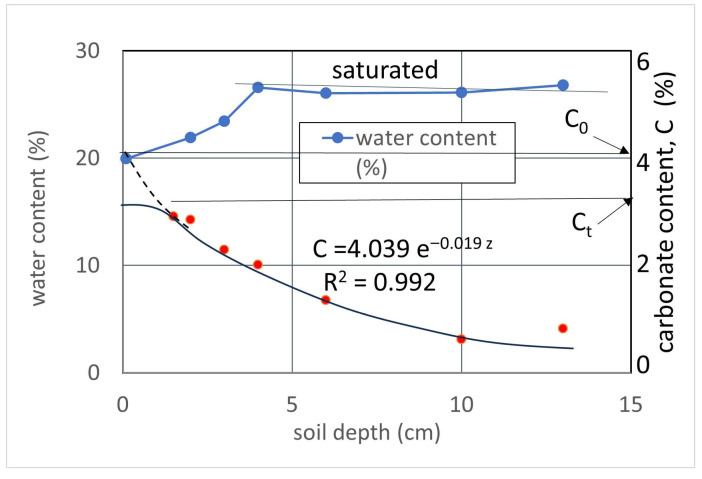
Test results of profiles of water contents and carbonate contents.

The water contents and carbonate contents were measured after more than three weeks after washing out the BCS. The water content test was made with the JIS (Japanese Industrial Standard), and the carbonate content was measured using a calcite-acid reactor [23]. The results of the water and carbonate contents are presented in terms of soil depth (Figure 7).

##### Water Content

Since the soil specimen was fine to medium sand, the porewater was retained well in the specimen. The specimen was left unattended for more than three weeks, but it remained almost saturated (higher than 25%) at a depth of 4 cm or more. On the other hand, the surface layer, 4 cm, was an unsaturated area, but the water content was at least 20%. Note that carbonate formation was completed in a few days, so that the carbonates were sufficiently produced before it became unsaturated. Accordingly, the retention rate Rr in Equation (13) should be 1.0, as mentioned earlier. The thickness of the soil sample was 135 mm.

##### Carbonate Precipitation in BCS

**Figure 8:**
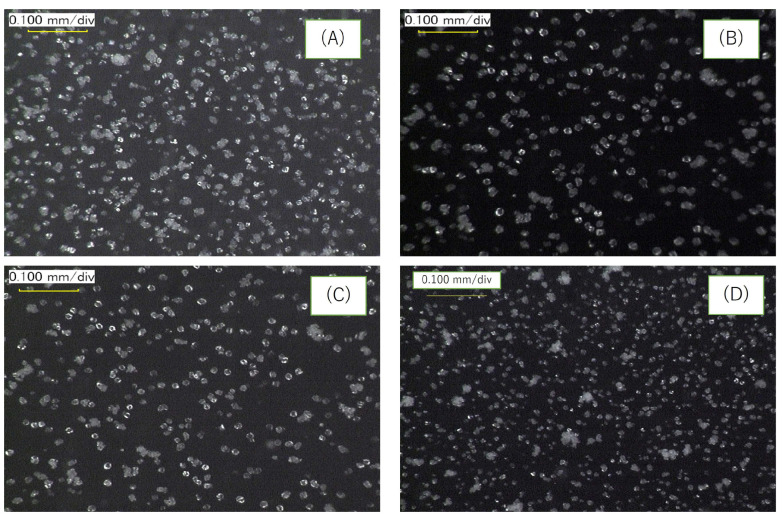
Images of carbonate particles precipitated in BCS used in the respective injection (with a digital microscope): (**A**) first injection, day 2; (**B**) second injection, day 2; (**C**) third injection, day 2; (**D**) fourth injection, day 2. The images show that there are dispersed aggregates of bacteria with ACC and/or calcites. The dispersion may be due to the negative charges of bacteria surfaces with ACC and/or calcites.

The carbonate precipitation in BCS was observed with a digital microscope, as shown in Figure 8. The Ca^2+^ concentration in each injection was 0.3 M except for the second injection. In the second injection, 0.4 M Ca^2+^ was used to see the difference between 0.3 and 0.4 M Ca^2+^. However, it was not clear except for a small difference in the particle aggregation. In this test, the total OD (as a representative value of the number of viable bacteria) was interesting because it provides information on the bacterial distribution.

During the infiltration of the designed BCS, the adsorption process in soils causes the dilution of the component of the BCS, which decreases the concentrations of bacteria and other solutes at a constant concentration ratio in the infiltrating BCS. As a result, the process was expressed by an exponential function with carbonate content. The C and soil depth relationship measured is demonstrated in Figure 7. The profile of carbonate content for the MICP-treated soil is presented as an exponential function, except for near the surface and the bottom of the specimen.

The function obtained is presented by two constants in a one-dimensional injection of BCS as

C = C_0_ exp (−R_a_ z) (%) (17)

where C_0_ is the ideal carbonate content at the surface of soils (z = 0) and R_a_ is the reduction rate of adsorption of carbonate content with depth. However, it is noted that the surface and bottom of the specimen are exceptional, depending on the boundary conditions. At the surface, the C_0_ is quite different from the actual carbonate content, which is often limited by many factors, such as the limit of adsorption or the disturbance of surface soil layer due to injection of BCS. The surface disturbance was seen for fine sand in MICP studies [36]. In Figure 7, the boundary conditions on such surfaces are expressed in C_t_. In this test, C_t_ was about 3.2%, but coincidentally, the carbonate content obtained was comparable to the total initial concentration of BCS injected.

The bottom provides a boundary condition associated with drainage types and the complicated flow pattern of BCS. As a result, the adsorption characteristics in terms of surface charge may be more complicated by the microscopic flow pattern and thus the interaction of bacteria, Ca^2+^, and soil particles.

In this result, it was found that the essence of the behavior of solutes adsorbed by microorganisms can be explained by the so-called adsorption theory to soils. This should be discussed in conjunction with the diffusion double layer theory [35].

##### Macro- to Micro-Properties in MICP

Prior to the injection, if the bacterial distribution in the BCS is equal and the Ca^2+^ diffuse double layers are similar for each bacterium, the number of bacteria in any of the aggregates after aggregation provides the potential carbonate content. The evidence can be shown by the OD-CPR relationship, as expressed later.

Therefore, it is assumed that the number of bacteria (OD) can control the values of CPR, C, and UCS. These relationships among the OD, CPR, C, and UCS are summarized as follows.

From Equations (12) and (14), Equation (18) can be obtained, as

CPR = C/W (M)(18)

Furthermore, from Equations (1) and (18):

OD = 0.118 C/W = 0.118 CPR(19)

Note that OD and CPR are a function of soil depth (z), and Equation (20) must be established.

C (z) = 8.46 W∙OD (z)(20)

Thus, the estimated CPR and OD are expressed as a function of C to be measured, as indicated in Equations (18) and (19). Therefore, as far as the OD controlled carbonate precipitation is used, the OD distribution can be estimated by the measured distribution of C. Accordingly, the distribution of bacteria after injection can be estimated by the C values. Then, the data to be obtained can be used for the study on adsorption properties and heterogeneity due to injection and/or the varied nature of soil properties.

Furthermore, this method may identify various types of back analysis used in geotechnology. Since the MICP technology can be applied for repair, rework, and repeated construction, it can be used in combination with back (reverse) analysis.

## 4. Discussion and Future Work

In this study, the basic issues were addressed in relation to MICP technology in general. Therefore, firstly, a series of studies was introduced, indicating that their adaptability and application are a challenge. Since the main material used is microorganisms, it is extremely difficult to handle them in the field of engineering, and much time is spent developing solutions. Through the manifestation and efforts of various issues, a solution was found. This section is a discussion of the solution, which has been done or is to be done. The following are selected issues and a brief discussion.

### 4.1. Bacterial Evaluation and Handling of Organisms as Engineering Materials

#### 4.1.1. Aging Problems in Bacteria

Not only urease-producing bacteria, but also cultured microorganisms, generally deteriorate due to changes in the surrounding environment, such as temperature, pH, concentration of bacteria, pressure, and existence of natural enemies. In general, the deterioration rate of bacteria is extremely fast at room temperature and refrigeration (see Figure 3). To keep the bacteria quality longer, ultra-low freezing temperatures should be used. In our studies, the temperature characteristics of bacteria were examined using the Rcv value, which was defined as viability [2,37].

#### 4.1.2. Chemical Reactions and Blending of BCS

The first chemical reaction of MICP is the hydrolysis of urea. However, there was no description of “How much bacteria are needed in the reaction?” In other words, until now, microorganisms have not been incorporated into the chemical reactions of MICP as official materials. This may be because the urease-producing bacteria have been realized as an enzyme which is not consumed. However, the bacteria become the nuclei of calcite formation and the roles of the enzyme are limited by the inhibition or retardation of Ca^2+^ [22]. Since the MICP process is strongly influenced by the Ca^2+^/OD, the treatment of OD becomes important in the MICP study.

### 4.2. Two Different Mechanisms in MICP (Ultimate CPRs)

In this study, optimal blending of BCS is considered, which was defined as Equation (1), i.e., CPR/OD = 8.46 M. For this blending, the ratio of CPR/OD can be sustained under dilution or concentration of BCS, as was assumed earlier, unless the materials in BCS cannot be separated. Therefore, it is assumed that the CPR/OD ratio can be sustained in soil pores during infiltration, unless CPR and OD (Ca^2+^ and bacteria) are independently separated.

As an ideal example, the adsorption of infiltrating aggregates (bacteria with Ca^2+^) onto soil particles seems to obey an adsorption theory. In that case, the measurements of C possibly become a key factor for the profiles of CPR, OD, and strength constants. If soil properties are inhomogeneous, the measurements of C will reflect the inhomogeneity of CPR and OD. In any case, irregularities related to the nature of the soil need to be dealt with as normal. The above assumption will be examined for future study.

### 4.3. Necessity of OD-Controlled Blending

In this study, the OD-controlled blending of BCS is recommended. Unless OD-controlled blending can be used in MICP technology, it is clear that various problems will arise. If it is not OD-controlled, it is impossible to estimate whether the carbonate precipitation is inhibited, the retardation is generated, or most of the microorganisms used are wasted [2,21,22]. It was revealed that the key to solving these problems is to determine and properly use the live bacterial rate.

### 4.4. Soil–BCS Systems

#### 4.4.1. Infiltration of BCS

There are many precautions regarding the infiltration of BC liquid. Many of them are due to the characteristics of the soil and the formulation of BCS, related to the resulting carbonate production. For example, soil permeability acts on the adsorption distribution of bacteria and solutes to the surface of soil particles in relation to the rate of infiltration of BCS. In addition, the particle size distribution of the soil is related to the specific surface area of the particles, which has a great influence on the adsorption distribution in addition to the infiltration rate. In general, BCS is prepared by mixing microorganisms with a reaction solution (including other solutes) at the end. The carbonate production reaction begins with the hydrolysis of urea in the cell immediately after the production of BCS, followed by the production of calcium carbonate outside the cell. Therefore, we needed to decide the starting time of the BCS infiltration. This flow process changes from amorphous calcium carbonate to crystal form rather slowly, but the details are more complicated, as follows. During infiltration of BCS, multiple physical and chemical actions occur. They are the formation of aggregates by electrostatic adsorption of microorganisms and ionic substances, the adsorption of aggregates on the surface of soil particles, and the amorphization or/and crystallization of aggregate–adsorbents [34,35,38,39,40]. Since the particle size of fine sand to gravel varies from about 0.063 to 75 mm, the specific surface area of particles also varies substantially in terms of soil type.

#### 4.4.2. Strength Development

In MICP technology, the prediction of soil strength development is not necessarily possible because there is no consensus on many points [41]. In essence, many of the issues raised in this study were not well understood. The most important challenge is that microorganisms have not been treated as engineering materials.

Except for the properties of microorganisms, the following three factors in terms of strength, carbonate content (C), unconfined compressive strength (UCS), and bearing capacity (Ru), should be understood. The UCS was examined by many researchers, but its utility has not necessarily been well established. With soil types, the size and texture of particles, and the initial dry density of soil should be taken into account in the design and construction [2]. Once these are done, it will be possible to predict the solidification strength, and the knowledge of geotechnical engineering will be applicable in the field of ground improvement. In particular, it will be possible to theoretically deal with the fracture and strength of solidified ground to design strength. For this reason, the approach in this study is extremely effective.

#### 4.4.3. Testing and Theory: Brittle Materials (Crystals)

The properties of MICP-solidified soil differ essentially almost from nearly zero to more than ten MPa. In other words, carbonate as a binding material for soil particles is crystalline, and its properties should be considered to be from weak glass to hard limestone.

The soil is mainly unconsolidated and loose sand. The desired solidification strength in the reduction in liquefaction potential is only approximately 100 kPa in UCS. A proper blending design is required to obtain the target unconfined compressive strength or other target strengths. In that case, solidification up to a few MPa will be wasted. However, it is not always easy to harden loose sand.

The strength determined by the unconfined compression test on MICP treatment on very loose sandy soils was extremely low in comparison to the uniaxial compressive strength estimated from the triaxial compression test. This is because the specimens fail by tensile forces and/or progressive destruction. However, when a slightly confined pressure is applied to the specimen, shear failure occurs, and the Mohr–Coulomb failure criterion can be applied [37]. In particular, when used as a method to reduce liquefaction, it is necessary to estimate the uniaxial compressive strength from vibration tests or triaxial compression tests.

## 5. Conclusions

After previous studies, the application of the knowledge obtained was examined in this study. The test results were evaluated comprehensively, and consensus was obtained. Firstly, the blending of BCS was examined using low-quality, aged bacteria and sufficient CPR was obtained qualitatively and quantitatively. However, the CPR resulted in a mass of carbonate that exceeded the amount of Ca^2+^ used. For now, it is only interpreted that the produced carbonate contains hydrates, but what were the conditions for this will be a question in the future. In fact, that calcite contains mono- and hexa-hydrates has been well known in nature. Therefore, there is no reason to ignore the precipitation of hydrate-calcites. There is also room for consideration as to whether hydrate has a significant effect on the engineering properties of the carbonate.

The challenge was how to adapt a low-quality, aged bacteria in the real field. It turns out that the principle of superposition can be applied to this. In other words, it was possible to solve the problem by increasing the concentration of bacteria. The Rcv value can be used for calculations and has the same effect as repeated injection.

The MICP treatment of fine medium sand samples under the ideal one-dimensional penetration conditions of BCS was performed to investigate the depth distribution of carbonate content C. As a result, it was revealed that ideal adsorption characteristics were exhibited except for the upper and lower parts of the specimen. In general, the surface layer often does not solidify due to structural disturbances due to osmosis, but this time, it seems to be the result of using an extremely slow laminar flow by intravenous drip.

In this study, we attempted to connect both microscopic and macroscopic perspectives. As a result, we were able to comprehensively express the relationship between carbonate content and BCS CPR using viable bacterial OD as the axis, taking into account the physical properties of the soil, in a single formula. This made it possible to estimate the distributions of CPR and OD from the measured *C* of soil.

In this study, the strength of treated soils was not investigated because it was known that the UCS can be primarily expressed by both C and the dry density of soil, and secondly by grain size distribution (uniformity) and textures of soil particles.

## Figures and Tables

**Table 1:** (**a**) Optimal-blending CPR values of BCS containing 0.25 M Ca^2+^ using aged, concentrated bacteria with low Rcv values (Rcv < 0.03) and (**b**) concentrated bacteria where Rcv = 0.3.

	No.	Date	Rcv	BCS	Assumed	Tempo.	Calc.	Meas.	Modified	CPR
				V_BCS_ (mL)	Rcv	OD*	V_BAC_ (mL)	V_BAC_ (mL)	OD*	(M)
**(a)**	50	19 June 2025	0.025	240	0.024	1.221	0.274	0.285	1.188	0.294
	83	16 June 2025	0.025	140	0.024	1.221	0.15	0.169	1.207	0.276
	16	12 June 2025	0.030	140	0.024	1.231	0.15	0.171	1.221	0.284
	4	10 June 2025	0.030	140	0.024	1.231	0.15	0.173	1.236	0.292
	3	6 June 2025	0.027	160	0.027	1.094	0.184	0.172	1.075	0.270
	7	5 June 2025	0.026	140	0.024	1.231	0.161	0.160	1.142	0.290
**(b)**	89	26 January 2025	0.300	240	0.300	0.100	0.024	0.024	0.100	0.281
	56	4 July 2025	0.300	240	0.300	0.100	0.024	0.029	0.121	0.270

**Table 2:** Conditions of BCS in infiltration tests.

Injection Interval from Start (d)	First 0	Second 2	Third 4	Fourth 7	Total
Ca^2+^ (M)	0.3	0.4	0.3	0.3	1.3
OD* A	0.3	0.31	0.35		
OD* B				0.31	
BCS (mL)	1000	1000	1000	1000	4000

## Data Availability

The original contributions presented in this study are included in the article. Further inquiries can be directed to the corresponding author.

## References

[1] Gollapudi U.K., Knutson C.L., Bang S.S., Islam M.R. (1995). A new method for controlling leaching through permeable channels. Chemosphere.

[2] Fukue M., Lechowicz Z., Mulligan C.N., Takeuchi S., Fujimori Y., Emori K. (2025). Properties and Behavior of Sandy Soils by a New Interpretation of MICP. Materials.

[3] Zhu T., Dittrich M. (2016). Carbonate Precipitation through Microbial Activities in Natural Environment, and Their Potential in Biotechnology: A Review. Front. Bioeng. Biotechnol..

[4] Bao S., Di J., Dong Y., Gao Z., Gu Q., Zhao Y., Zhai H. (2024). Experimental study on the effect of cementation curing time on MICP bio-cemented tailings. Constr. Build. Mater..

[5] Mark J., Jones M.J., Butchins L.J., Charnock J.K., Pattrick R.A.D., Small J.S., Vaughan D.J., Wincott P.L., Livens F.R. (2011). Reactions of radium and barium with the surfaces of carbonate minerals. Appl. Geochem..

[6] Meng H., Gao Y., He J., Qi Y., Hang L. (2021). Microbially induced carbonate precipitation for wind erosion control of desert soil: Field-scale tests. Geoderma.

[7] Achal V., Pan X., Zhang D. (2012). Bioremediation of strontium (Sr) contaminated aquifer quartz sand based on carbonate precipitation induced by Sr resistant *Halomonas* sp.. Chemosphere.

[8] Chen M., Li Y., Jiang X., Zhao D., Liu X., Zhou J., He Z., Zheng C., Pan X. (2021). Study on soil physical structure after the bioremediation of Pb pollution using microbial-induced carbonate precipitation methodology. J. Hazard. Mater..

[9] Dong Y., Gao Z., Di J., Wang D., Yang Z., Wang Y., Guo X., Li K. (2023). Experimental study on solidification and remediation of lead–zinc tailings based on microbially induced calcium carbonate precipitation (MICP). Constr. Build. Mater..

[10] Zhao J., Csetenyi L., Gadd G.M. (2022). Fungal-induced CaCO_3_ and SrCO_3_ precipitation: A potential strategy for bioprotection of concrete. Sci. Total Environ..

[11] Han L., Li J., Xue Q., Chen Z., Zhou Y., Poon C.S. (2020). Bacterial-induced mineralization (BIM) for soil solidification and heavy metal stabilization: A critical review. Sci. Total Environ..

[12] Hu L., Wang H., Xu P., Zhang Y. (2021). Biomineralization of hypersaline produced water using microbially induced calcite precipitation. Water Res..

[13] Li M., Cheng X., Guo H. (2013). Heavy metal removal by biomineralization of urease producing bacteria isolated from soil. Int. Biodeterior. Biodegrad..

[14] Lin H., Zhou M., Li B., Dong Y. (2023). Mechanisms, application advances and future perspectives of microbial-induced heavy metal precipitation: A review. Int. Biodeterior. Biodegrad..

[15] Yang Z., Zhang Z., Sun L., Yuan L. (2022). Characteristics and possible formation process of ferromanganese beachrock in an intertidal zone of East Chaina Sea influenced by buried ancient woods. Mar. Geol..

[16] Zhang X., Wang H., Wang Y., Wang I., Cao J., Zhan G. (2025). Improved methods, properties, applications and prospects of microbial induced carbonate precipitation (MICP) treated soil: A review. Biogeotechnics.

[17] Payan M., Sangdedh M.K., Salimi M., Ranjbar P.Z., Arabani M., Hosseinpour I. (2024). A comprehensive review on the application of microbially induced calcite precipitation (MICP) technique in soil erosion mitigation as a sustainable and environmentally friendly approach. Result Eng..

[18] Maston O., Ouahbi T., Taibi S., Hajjar A.E., Sapin L. (2025). Effect of MICP treatment on the mechanical properties of clay soils. Transp. Geotech..

[19] Alam M.K., Motamed R. (2026). Role of MICP treatment area in mitigating liquefaction-induced settlements for shallow foundation. Biogeotechnics.

[20] Omoregie A.I., Palombo E.A., Nissom P.M. (2021). Bioprecipitation of calcium carbonate mediated by ureolysis: A Review. Environ. Eng. Res..

[21] Fukue M., Lechowicz Z., Fujimori Y., Emori K., Mulligan C.N. (2022). Incorporation of optical density into the blending design for a biocement solution. Materials.

[22] Fukue M., Lechowicz Z., Fujimori Y., Emori K., Mulligan C.N. (2023). Inhibited and Retarded Behavior by Ca^2+^ and Ca^2+^/OD Loading Rate on Ureolytic Bacteria in MICP process. Materials.

[23] Fukue M., Ono S., Sato Y. (2011). Cementation of sands due to Microbiologically induced Carbonate. Soils Found..

[24] Lai H.-J., Cui M.-J., Wu S.-F., Yang Y., Chu J. (2021). Retarding effect of concentration of cementation solution on biocementation of soil. Acta Geotech..

[25] Cui M.-J., Teng A., Chu J., Cai B. (2022). A quantitative, high-throughput urease activity assay for comparison and rapid screening of ureolytic bacteria. Environ. Res..

[26] Song H., Kumar A., Zhang Y. (2022). Microbial-induced carbonate precipitation prevents Cd^2+^ migration through the soil profile. Sci. Total Environ..

[27] Bachmeier K.L., Williams A.E., John R., Warmington J.R., Bang S.S. (2002). Urease activity in microbiologically-induced calcite precipitation. J. Biotechnol..

[28] Mobley H.L.T., Hausinger R.P. (1989). Microbial, urease: Significance, regulation, and molecular characterization. Microbiol. Rev..

[29] Zambare N.M., Naser N.Y., Gerlach R., Chang C.B. (2020). Mineralogy of microbially induced calcium carbonate precipitates formed using single cell drop-based microfluidics. Sci. Rep..

[30] Arai Y., Yasue T. (1981). Crystal Structure of Calcium Carbonates and Their Hydrates. Gypsum Lime.

[31] Perry T.D., Cygan R.T. (2007). Ralph Mitchell, Molecular models of a hydrated calcite mineral surface. Geochim. Cosmochim. Acta.

[32] Page K., Stack A.G., Chenc S.A., Wang H.-W. (2022). Nanopore Facilitated Monohydrocalcitic Amorphous Calcium Carbonate Precipitation. Phys. Chem. Chem. Phys..

[33] Freeman C.L., Harding J.H. (2023). The transformation of amorphous calcium carbonate to calcite and classical nucleation theory. J. Cryst. Growth.

[34] Yee N., Fein J.B., Daughney C.J. (2000). Experimental study of the pH, ionic strength, and reversibility behavior of bacteria–mineral adsorption. Geochim. Cosmochim. Acta.

[35] Poortinga A.T., Bos R., Norde W., Busscher H.J. (2002). Electric double layer interactions in bacterial adhesion to surfaces. Surf. Sci. Rep..

[36] van Paassen L. (2009). Biogrout, Ground Improvement by Microbially Induced Carbonate Precipitation. Ph.D. Thesis.

[37] Mulligan C.N., Fukue M., Yong R.N. (2025). Sustainable Practices Geoenvironmental Engineering.

[38] Shen X., Zheng X., Bourg I.C. (2025). A coarse-grained model of clay colloidal aggregation and consolidation with explicit representation of the electrical double layer. J. Colloid Interface Sci..

[39] Baciocchia R., Bonib M.R.B., Lavecchia R. (2005). Modeling of chlorophenols competitive adsorption on soils by means of the ideal adsorbed solution theory. J. Hazard. Mater..

[40] Zhao Y.-Y., Zhang M., Hu X.-M., Feng Y., Xue D., Wang Q.-S., Geng Z., Liu Y., Zhang J., Jia X.-H. (2023). Study on adsorption and dust suppression mechanism of urease-producing bacteria on coal-soil mixed dust. J. Environ. Chem. Eng..

[41] Konstantinou C., Wang Y., Biscontin G., Soga K. (2021). The role of bacterial urease activity on the uniformity of carbonate precipitation profiles of bio-treated coarse sand specimens. Sci. Rep..

